# Supplementary material for: Repetitive Sequence Transcription in Breast Cancer
Source: Cells. 2022 Aug 14;11(16):2522. doi: 10.3390/cells11162522 (PMC9406339; doi:10.3390/cells11162522)
Supplement: Supplementary file 1 [file cells-11-02522-s001.zip › supplementary/Webpage_S4_Deseq2Webpage.pdf]

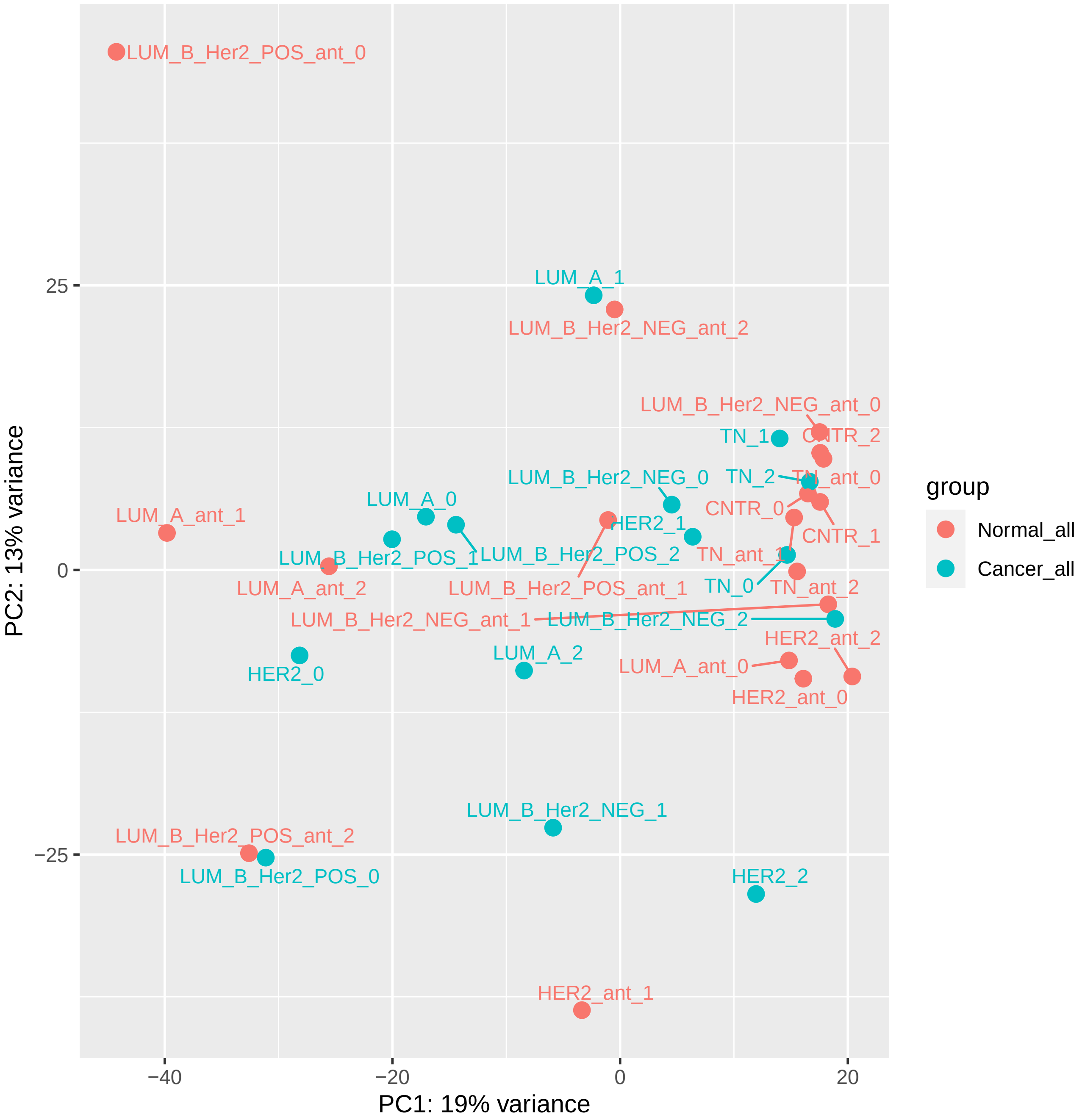
LUM_B_Her2_POS_ant_0
LUM_A_1
LUM_B_Her2_NEG_ant_2
LUM_B_Her2_NEG_ant_0
TN_1
CNTR_2
LUM_B_Her2_NEG_0
TN_2
TN_ant_0
LUM_A_0
CNTR_0
LUM_A_ant_1
HER2_1
CNTR_1
LUM_B_Her2_POS_1
LUM_B_Her2_POS_2
TN_ant_1
TN_0
TN_ant_2
LUM_A_ant_2
LUM_B_Her2_POS_ant_1
LUM_B_Her2_NEG_ant_1
LUM_B_Her2_NEG_2
HER2_ant_2
LUM_A_2
LUM_A_ant_0
HER2_0
HER2_ant_0
LUM_B_Her2_NEG_1
LUM_B_Her2_POS_ant_2
LUM_B_Her2_POS_0
HER2_2
HER2_ant_1
25
0
−25
−40
−20
0
20
PC1: 19% variance
PC2: 13% variance
group
Normal_all
Cancer_all

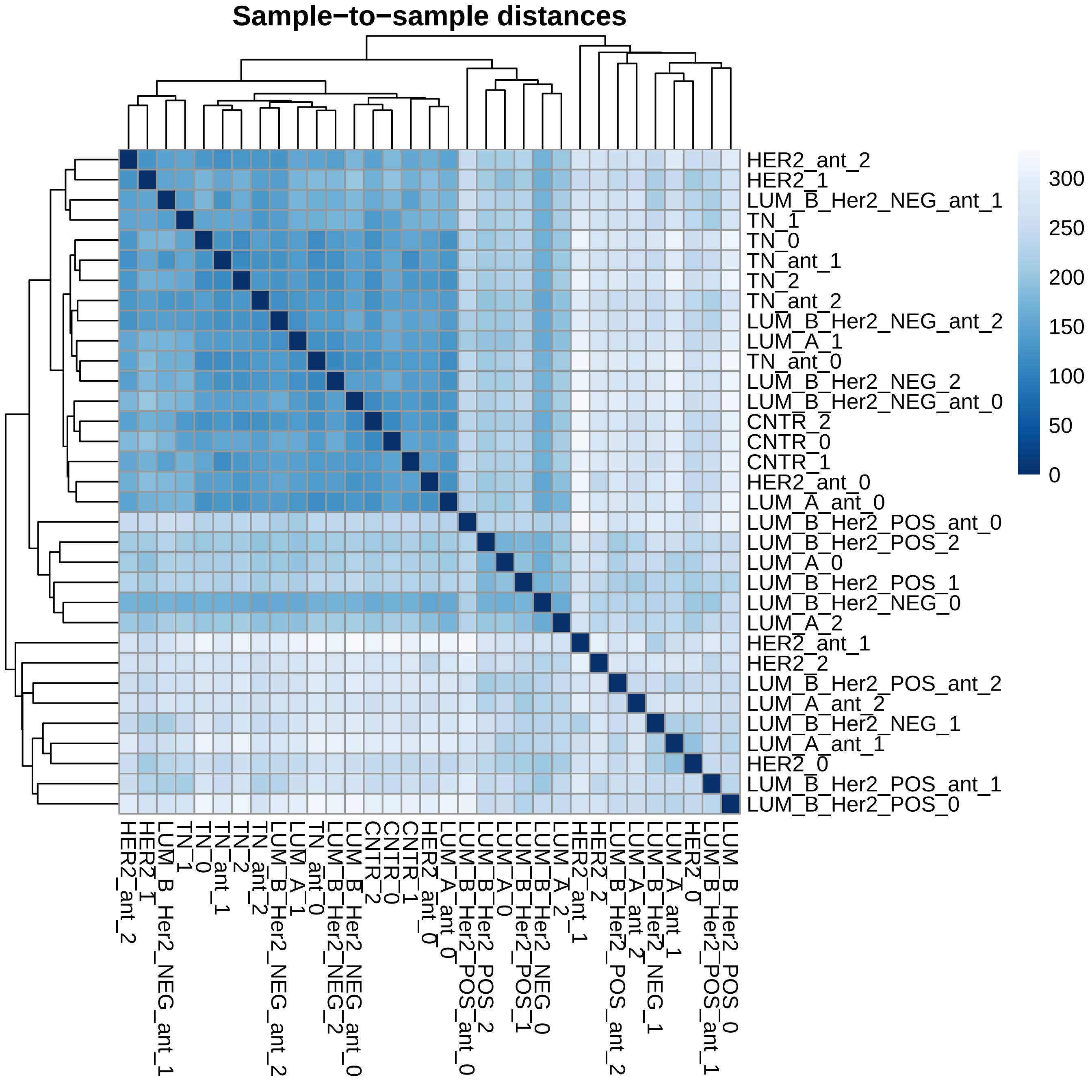
Sample−to−sample distances
HER2_ant_2
HER2_1
LUM_B_Her2_NEG_ant_1
TN_1
TN_0
TN_ant_1
TN_2
TN_ant_2
LUM_B_Her2_NEG_ant_2
LUM_A_1
TN_ant_0
LUM_B_Her2_NEG_2
LUM_B_Her2_NEG_ant_0
CNTR_2
CNTR_0
CNTR_1
HER2_ant_0
LUM_A_ant_0
LUM_B_Her2_POS_ant_0
LUM_B_Her2_POS_2
LUM_A_0
LUM_B_Her2_POS_1
LUM_B_Her2_NEG_0
LUM_A_2
HER2_ant_1
HER2_2
LUM_B_Her2_POS_ant_2
LUM_A_ant_2
LUM_B_Her2_NEG_1
LUM_A_ant_1
HER2_0
LUM_B_Her2_POS_ant_1
LUM_B_Her2_POS_0
300
250
200
150
100
50
0

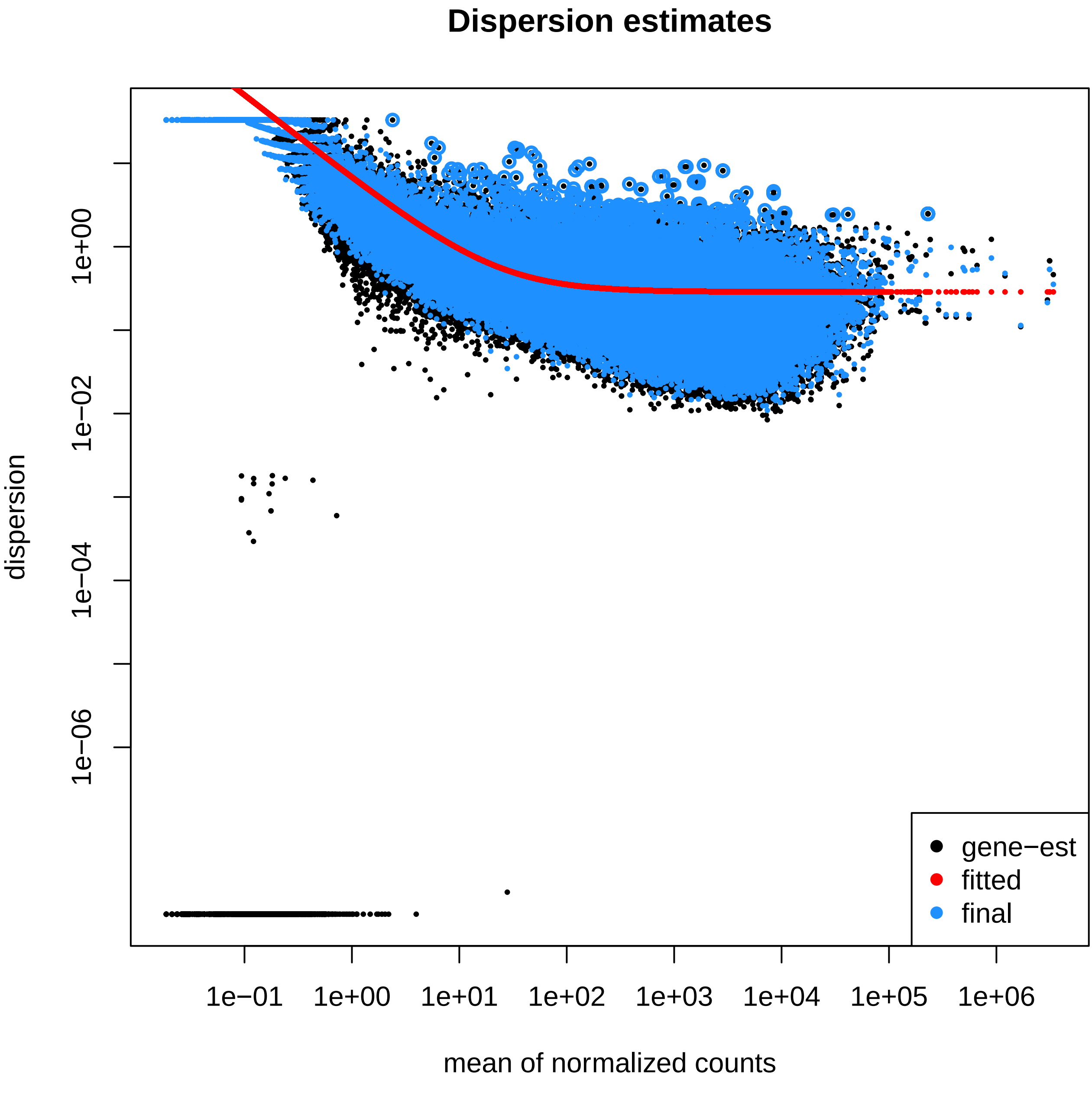
Dispersion estimates
dispersion
1e+00
1e−02
1e−04
1e−06
1e−01
1e+00
1e+01
1e+02
1e+03
1e+04
1e+05
1e+06
mean of normalized counts
gene−est
fitted
final

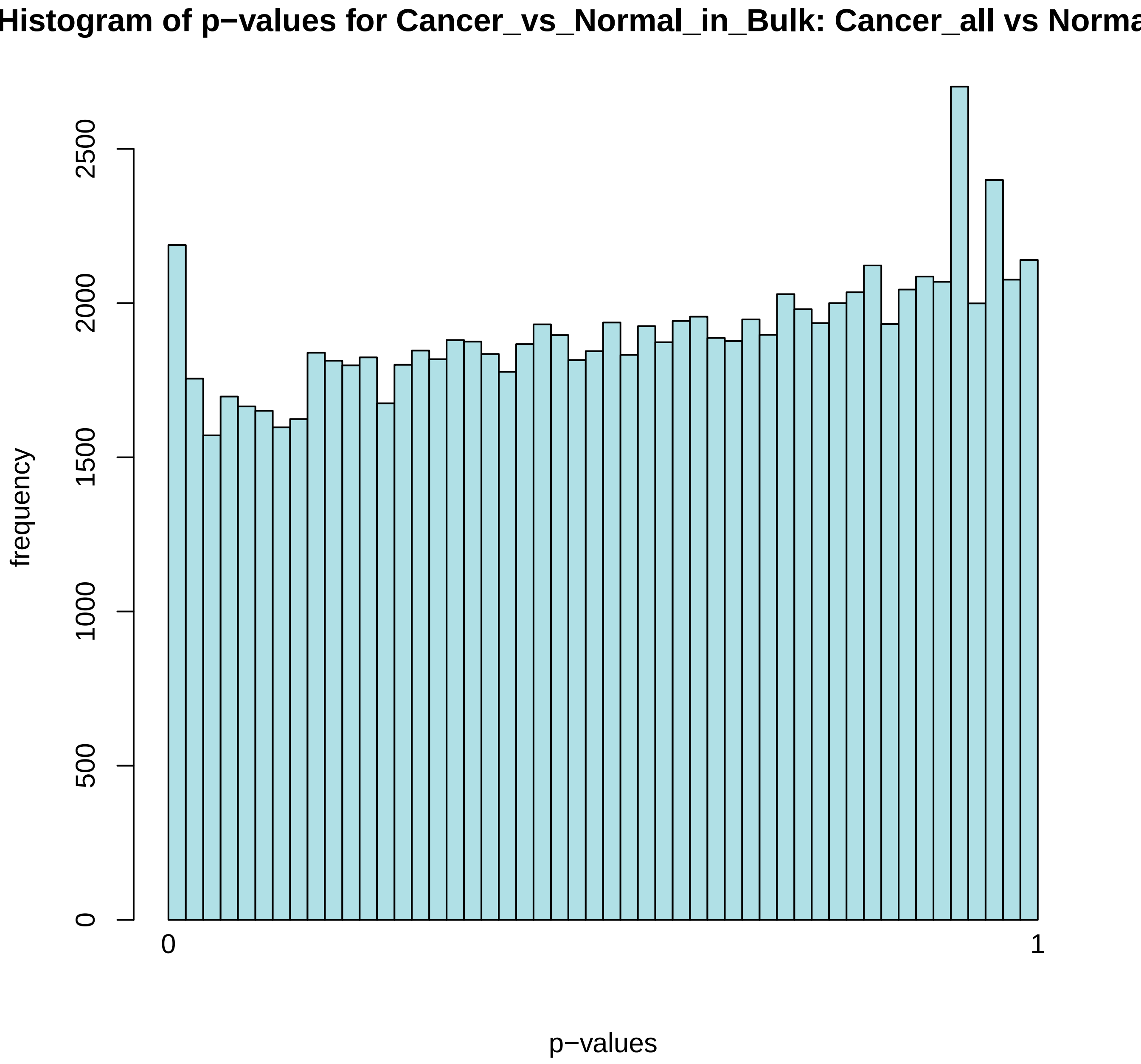
Histogram of p−values for Cancer_vs_Normal_in_Bulk: Cancer_all vs Norma
frequency
0
500
1000
1500
2000
2500
0
1
p−values

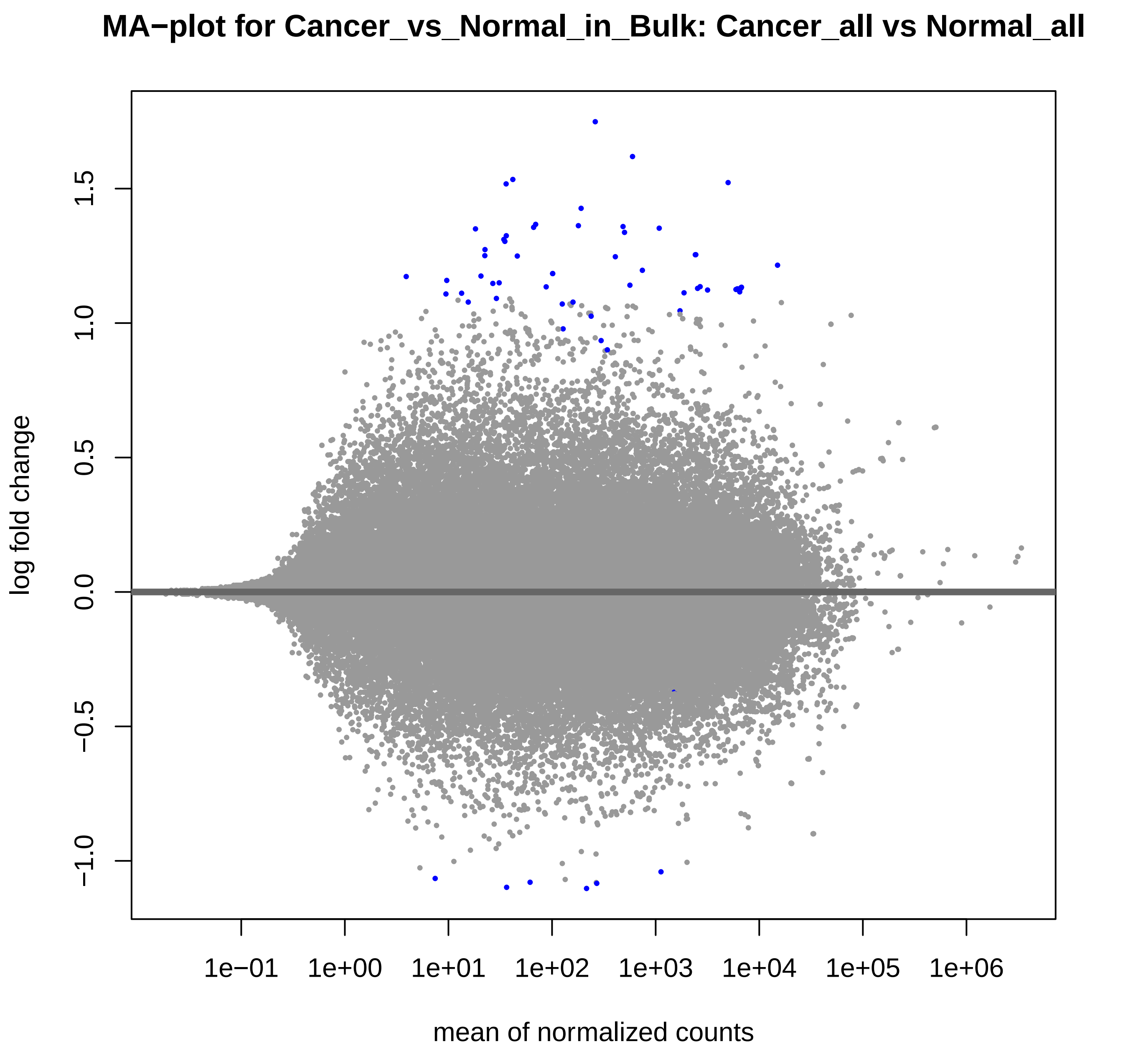
MA−plot for Cancer_vs_Normal_in_Bulk: Cancer_all vs Normal_all
log fold change
1.5
1.0
0.5
0.0
−0.5
−1.0
1e−01
1e+00
1e+01
1e+02
1e+03
1e+04
1e+05
1e+06
mean of normalized counts
